# Future Perspectives in the Management of Otitis Media

**DOI:** 10.3390/medicina59091553

**Published:** 2023-08-26

**Authors:** Andrea Frosolini, Andrea Lovato

**Affiliations:** 1 Maxillofacial Surgery Unit, Department of Medical Biotechnologies, University of Siena, 53100 Siena, Italy; andreafrosolini@gmail.com; 2 Otorhinolaryngology Unit, Department of Surgical Specialties, Vicenza Civil Hospital, 36100 Vicenza, Italy

Otitis media, which encompasses acute otitis media (AOM) and chronic otitis media (COM), is a prevalent and significant health issue affecting both children and adults [1]. This condition can have a profound impact on individuals’ quality of life, leading to discomfort, pain, hearing loss, and a plethora of local and systemic complications [2]. Moreover, the costs associated with medical consultations, medications, surgeries, and hearing rehabilitation can be substantial for individuals, families, and healthcare systems. The papers included in this Special Issue of *Medicina* contribute to our understanding of the pathophysiology of otitis media, identify risk factors, evaluate treatment approaches, and explore innovative strategies for prevention and treatment. We received numerous submissions, and after a rigorous peer-review process, we are pleased to present the findings of four accepted papers that cover a range of topics related to otitis media and provide valuable insights into this condition [3,4,5,6].

Otitis media in the pediatric population represents a concerning issue for clinicians. The paper entitled “Watchful Waiting in Paediatric Acute Otitis Media: A Real Practice Approach or an Intangible Desideratum?” by Spoială et al. [3] provides an in-depth analysis of antibiotic prescribing patterns for acute otitis media in children [3]. This comprehensive review of international literature published in recent years reveals variations in antibiotic prescription rates and emphasizes the need for judicious use of antibiotics, promoting the implementation of watchful waiting strategies. The paper entitled “Temporomandibular Joint and Otitis Media: A Narrative Review of Implications in Etiopathogenesis and Treatment” by Bernkopf et al. [4] presents a comprehensive review of the relationship between temporomandibular joint dysfunction and otitis media. This article highlights the role of a dysfunctional stomatognathic system in the etiopathogenesis of otitis media and discusses the effectiveness of orthognathic treatment in preventing recurrent otitis media and otitis media with effusion [5]. The paper entitled “Resolution of Otitis Media with Effusion in Adults after a Three-Day Course of Treatment with a Manosonic Nebulizer—A Pilot Study” by Zasadzińska-Stempniak et al. [5] presents the findings of a pilot study evaluating the efficacy of an automatic manosonic aerosol generator in treating otitis media with effusion (OME) in adults [5]. The results show promising outcomes, although further research is needed to validate these findings. Lastly, the paper entitled “Chronic Otitis Media in Patients with Chronic Rhinosinusitis: A Systematic Review” by Brescia et al. [6] examines the international literature, according to the PRISMA guidelines, regarding the association between COM and chronic rhinosinusitis. This review identifies evidence that chronic rhinosinusitis is significantly associated with COM in the context of a global inflammatory process that involves the epithelium in both the middle ear and upper airway [6]. The recognition of this relationship may contribute to preventing chronic inflammatory conditions through early management.

We believe that these papers offer valuable insights and advancements to the field of otitis media diagnosis and treatment. The findings presented in this Special Issue have the potential to improve patient outcomes and guide future research. To date, one notable area of investigation is the role of probiotics in preventing acute and chronic otitis media. Some proposed mechanisms of action for probiotics include (i) stabilizing microbiota of the nasopharynx and middle ear; (ii) maintaining epithelial cell barrier function; (iii) modulating and enhancing immune function; (iv) competing for nutrients and adhesion sites on the surfaces of epithelial cells with pathogenic bacteria; and (v) producing bacteriocins and other inhibitory substances [7]. Recent studies have highlighted the preventive potential of probiotics: a systematic review of 17 randomized controlled trials (RCTs) shows that probiotics are associated with a lower proportion of children experiencing one or more episodes of AOM during treatment, indicating a potential preventive effect of probiotics. Probiotics have also led to a decrease in the proportion of children taking antibiotics for AOM [8]. Since the best management for OME has yet to be defined and recent international guidelines strongly recommended against the use of steroids, antibiotics, and antihistamine drugs, research on effective treatments for improving Eustachian tube function represents an active field of investigation [5,9]. Recently, there is emerging evidence suggesting that dental malocclusion treatment can play a role in preventing otitis media [10], as well as complications of otitis media can affect the temporomandibular joint [11]. In this context, the management of otitis media in individuals with craniofacial dysmorphism continues to be a topic of debate [12]. In both practical application and scientific research, there are debates about the management of COM, particularly tympanoplasty, concerning potential diverse surgical approaches and applications of various available materials [13,14]. In the field of hearing rehabilitation, there have been exciting developments in osseointegrated bone-anchored hearing aids as well as in non-implantable devices; therefore, these prosthetic devices are profitably applied in COM patients at present [15].

We would like to express our gratitude to all the authors who submitted their work to this Special Issue, and we also extend our appreciation to the reviewers for their valuable feedback and contributions in ensuring the high quality of the published papers. To conclude this Editorial, we encourage readers to explore the diverse topics covered and consider their implications for clinical practice. By disseminating this knowledge and promoting evidence-based medicine, we can work toward more effective prevention, early intervention, and comprehensive management of otitis media. Ultimately, this will lead to better outcomes for our patients by improving their overall health and well-being.
